# The impact of government subsidy and weather on environmentally sustainable investment decision for agricultural supply chain

**DOI:** 10.1371/journal.pone.0285891

**Published:** 2023-05-18

**Authors:** Hongyong Fu, Ting Zhou, Shuguang Zhang, Qi Wang

**Affiliations:** 1 Research Center of Supply Chain Compliance Management, Southwest University of Political Science and Law, Chongqing, China; 2 Department of Modern Logistics, National University of Singapore (Chongqing) Research Institute, Chongqing, China; University of Naples Federico II: Universita degli Studi di Napoli Federico II, ITALY

## Abstract

This paper studies the environmentally sustainable investment of an agricultural supply chain composed of a farmer and a company, under three subsidy policies which are the non-subsidy policy, the fixed subsidy policy, and the Agriculture Risk Coverage (ARC) subsidy policy. Then, we analyse the impact of different subsidy policy and adverse weather on the costs of the government and profits of the farmer and the company. By comparing with the non-subsidy policy, we find that both the fixed subsidy policy and the ARC policy encourage the farmer to improve the environmentally sustainable investment level and increase the profit of the farmer and the company. We also find that both the fixed subsidy policy and the ARC subsidy policy lead to an increase in government spending. Our results show that the ARC subsidy policy has a significate advantage in encouraging the farmer’s environmentally sustainable investment if the adverse weather is relatively serious, comparing with the fixed subsidy policy. In turn, our results also show that the ARC subsidy policy is more beneficial for both the farmer and the company than the fixed subsidy policy if the adverse weather is relatively serious, which then leads to a higher expenditure of the government. Therefore, our conclusion serves as a theoretical basis for governments to formulate agricultural subsidy policies and promote sustainable development of the agricultural environment.

## 1. Introduction

As a basic industry, agriculture plays an important role in supporting industrial production and the healthy development of service sectors, and ensuring national security. As for developing countries, taking China as an example, the government has placed three rural issues (agriculture, rural areas, and farmers) into national strategies. Over the past 15 years (2004–2018), China has implemented a policy document dubbed as No.1 Central Document and a series of policies to benefit farmers [1]. As for developed countries, taking the United States as an example, the data from the Department of Agriculture (USDA) in 2013 showed that, agriculture and agriculture-related business contributed $789 billion to its GDP [2]. Overall, food, beverage, clothing, and catering sectors rely on agricultural inputs and outputs to create added value.

However, agriculture has proven to be one of the largest polluting industries in the world [3]. Searchinger et al. [4] also point out that a quarter of the greenhouse gas emissions produced annually are caused by agricultural operations and land use change. To make matters worse, global food demand is currently increasing rapidly, which is a huge challenge for the farmers in environmentally sustainable investment. Generally, how to guarantee global food security by reducing environmental costs while increasing yields has become a pressing reality.

In the first place, uncertainty is a typical feature of agriculture, which affects its sustainable and healthy development. The uncertainty is mainly in outputs [5, 6], purchasing prices [7], demand [8, 9] of agricultural products as well as food safety problem, and the most typical one is output. Diseases and pests [10] and adverse weather [11] (warm winter, late spring coldness, etc.) are the two core factors affecting the outputs of agricultural products. While pests and diseases could be partially controlled, adverse weather is artificially uncontrollable in agricultural production. At the same time, the uncertainty in outputs of agricultural products affects the income of farmers [2] and agriculture-related downstream companies in the agricultural supply chain [11], which should also be taken into consideration. Therefore, this paper, taking warm winter as an example, studies the increase of farmers’ income and environmentally sustainable operation of the agricultural supply chain under the impact of uncertainty of agricultural outputs. However, All of our results can be applied to the other adverse weather, such as drought and flood disaster. Also, the sound development of agriculture should balance the economic benefit of farmers and environmental protection [12, 13]. For example, Barilla of Italy encouraged farmers to produce in an environmentally sustainable way by providing farmers with PGP (partially-guaranteed-price) contracts, and it has reduced 30% of carbon emissions [14].

Secondly, to alleviate fluctuations in the income of farmers and agriculture-related companies caused by uncertainty and to ensure the sustainable development of the agricultural environment, governments of all countries have issued a series of agricultural subsidy policies [15]. For instance, every five years or so, the US congress upgrades comprehensive legislation on agricultural projects; each year, China, a representative of developing countries, releases the No.1 Central Document on agricultural subsidy. Currently, there are two typical subsidy policies in the world. One is the fixed subsidy policies, implemented by China, a developing country [16]; the other one is the ARC subsidy policy, applied by the United States, a developed country [2].

- *Fixed subsidy policy*—Under this policy, farmers are paid by a fixed subsidy for each kilogram of agricultural product, especially grain production, i.e. production-quantity-based subsidy, which is equal to price support and changes the price received for grain output [16].
- *Agriculture Risk Coverage (ARC) subsidy policy—* Under this policy, farmers receive a subsidy when their crop revenue in a given year drops below a reference revenue point which is based on a multiyear moving average of historical crop revenue [2].

The emphasis of all governments on agricultural subsidy has driven scholars to study the sustainable operation of the agricultural supply chain with government subsidy. Such study requires a better understanding of the government subsidy mechanism and the impact of government subsidy on every stakeholder. Government subsidy plays an important role in the sound development of agriculture, in both developing and developed countries. Tang et al. [17] study the impact of market information and agricultural policies on farmers in developing economies and imply the role of government subsidy in the agricultural development of developing countries. Alizamir et al. [2] study the significance of government subsidies in agricultural development in developed countries, and first conduct a quantitative study of the impact of government subsidies on the performance of stakeholders. They point out that future research should consider weather conditions as the main factor affecting agricultural outputs, and that the quantitative study should be done to measure the impact of government subsidies on dealing with adverse weather. Based on the analysis above, this paper, taking adverse weather into consideration, studies the impact of government subsidies on stakeholders in the agricultural supply chain under the impact of adverse weather. The following questions are expected to be answered.

1. How do the fixed subsidy policy and the ARC subsidy policy affect the environmentally sustainable investment decided by farmers in the adverse weather of warm winter?
2. How do the fixed subsidy policy and the ARC subsidy policy affect the incomes of farmers and companies and the expenditures of the government under the adverse weather?
3. What is the executable condition where the fixed subsidy policy is more beneficial for farmers than the ARC subsidy policy? And, the condition where the ARC subsidy policy is more beneficial for farmers than the fixed subsidy policy?

### 1.1 Summary of results

Based on the above model characteristics, our analysis reveals the following results. Firstly, we show that comparing with the case no subsidy provided by the government, both the fixed subsidy policy and the ARC subsidy policy can encourage the farmer in environmentally sustainable investment, which in turn benefit both the farmer and the company. We also show that all the two subsidy polices can weaken the adverse effects from the adverse weather.

Secondly, by comparing the environmentally sustainable investment under fixed subsidy policy with that under ARC subsidy policy, we find that the performance of the ARC subsidy policy in encouraging environmentally sustainable investment is better than that of the fixed subsidy policy if the adverse weather is relatively serious, vice versa.

Thirdly, our results show that the profits of the farmer and the company under the ARC subsidy policy is greater than those under the fixed subsidy policy if the adverse weather is relatively serious, vice versa. This is in line with the impact of different subsidy policy on encouraging environmentally sustainable investment. We also find that the government should pay more subsidy to the government under the ARC subsidy policy if the adverse weather is relatively serious.

The rest of this study is organized as follows. In section 2, we summarize the related literature. We provide the details of our research problem and model setup in Section 3. In Section 4, we analyze the equilibrium decisions for environmentally sustainable investments under no subsidy policy, fixed subsidy policy and ARC subsidy policy, respectively. In Section 5, we provide a two-by-two comparative analysis of the three subsidies to explore the implementation effects of the different subsidy policies. Finally, In Section 6, we conclude the paper with a summary of the results.

## 2. Literature review

In this paper, our work contributes to the literature that researches farmers’ optimal decisions under uncertainty, such as yield uncertainty, demand uncertainty, wholesale price uncertainty, and uncertain protection schemes that are designed to protect farmers and support farming. In agriculture economics, Sherrick et al. [18] and Coble et al. [19] investigate the relationship between the protection schemes and insurance, futures, and options. However, the subsidies formulation analyzed in these papers is different from the fixed subsidy and ARC subsidy. Most importantly, they do not build an analytical model to study the impact of subsidy schemes on stakeholders in the agricultural supply chain. Although recent publication builds an analytical model to examine the impacts of PLC subsidy and ARC subsidy on farmers’ profits [2], company’s profits, and government expenditures, we are not aware of any analytical model that studies government subsidy to protect farmers’ income against adverse weather-sensitive yield uncertainty.

Our work is closely related to four streams of research, which are the agricultural operations management literature under uncertain yield, the management of agricultural operations in developing economies, Sustainable agricultural supply chain and the government subsidy under different contexts. These studies are briefly reviewed below.

Firstly, yield uncertainty is a characteristic of the agricultural supply chain. Our work contributes to the literature on agricultural operations management that investigates the impacts of uncertain yield on stakeholders’ decisions in the supply chain. Kazaz [20] assumes that the cost of purchasing olives and the retail price are exogenous and decreasing in output, then studies production planning with random yield in the olive oil industry. Kazaz and Webster [21] investigate the optimal selling price and production quantity under the impact of yield-dependent trading cost. Boyabatli et al. [10] study the processing and storage capacity investment and periodic inventory decisions under yield uncertainties. However, these papers do not address subsidy mechanisms from the government. Moreover, these papers also do not consider the source of uncertain yield. The yield uncertainty is mostly caused by crop diseases and pests [22], or by uncontrollable adverse weather (such as mild winter, cold spells in late spring, etc.) [11]. Compared with to some extent controllable crop diseases and pests, uncontrollable adverse weather must be taken into consideration in agricultural supply chain management, due to the fact that climate risks have a significant negative impact on supply chain participants’ performance in the agricultural supply chain [23] and further make agricultural production face greater uncertainty and increase farmers’ economic losses [24]. It is, therefore, necessary to study agricultural operations management with weather-related yield under government subsidies.

Secondly, our work contributes to the growing body of research on agricultural operations management in developing countries or regions. Some experts and scholars study water resource management and the impact of weather on farmers’ decisions in developing economies. Dawande et al. [25] propose decentralized and individually rational mechanisms to achieve socially optimal distribution of surface water for a farming community under uncertainty in rainfall in India and show that the mechanisms can be efficiently computed. We also study the movement of the price of water with its scarcity. Ideas that can help administer the mechanisms in practice are briefly discussed. Huh and Lall [26] study a decision problem of a farmer associated with allocating his land among different crops with varying water requirements. Murali et al. [27] research a municipal groundwater management problem to determine optimal allocation and control policies for water transfer opportunities. However, no existing studies study cooperation among farmers in the agricultural supply chain. Specifically, An et al. [28] examine different effects of aggregating farmers through cooperatives. Chen et al. [29] investigate the effectiveness of peer-to-peer interactions among farmers. These two papers do not consider the cooperation of farmers and companies and the impact of government subsidies on the agricultural supply chain. Tang et al. [17] show that the government should consider offering subsidies to reduce the upfront investment cost in developing economies. However, they do not model government subsidies and examine the impact of the subsidy on sustainable development in the agricultural supply chain.

Thirdly, sustainable agricultural supply chain (SASC) management has been a topic of high interest to scholars and practitioners in recent years. Agricultural sustainability goals were highlighted as early as the UN 2030 Agenda, however, uncertainty of market demand and farming methods highly dependent on weather and rainfall conditions further aggravate the complexity of sustainable agricultural supply chain problems [30]. Excessive use of synthetic fertilizers and pesticides also has negative environmental impacts and it is vital to find environmentally sustainable ways to grow more food. Chai et al. [31] find that integrated intercropping agriculture could increase food production while reducing the environmental footprint. Smallholder farmers in China often tend to overuse nitrogen fertilizer as an ’insurance policy’ against yield loss, but this practice can lead to serious environmental pollution [32]. Based on this, Yin et al. propose a long-term steady-state nitrogen balance (SSNB) approach that not only significantly reduces N fertilizer use, but also increases yields and contributes to the sustainability of agriculture [32], while Searchinger et al. [4] argue that reducing food losses is a profound solution to creating a sustainable food future. Cui et al. [33] suggest that building trust, participatory innovation, developing human capacity, and strengthening the coherence of farming communities play a crucial role in the sustainability of agriculture. Moreover, Ren et al. [34] find that rapid population ageing leads to increased fertilizer losses and exacerbates the release of environmental pollutants, and that the management of rural ageing will contribute to the overall transition of smallholder farmers to sustainable agriculture in China. Wang et al. [35] suggest that an integrated knowledge and product strategy (IKPS) approach can improve food security while reducing resource and environmental burdens, and Duan et al. [36] find that the integration of agricultural land can contribute to sustainable agricultural development in China. In conclusion, the negative environmental impacts of agricultural activities can be curbed by improving nutrient use efficiency, increasing nutrient recovery, reducing food waste, increasing food production, reducing greenhouse gas emissions, and increasing agricultural productivity to promote the practice of sustainable agricultural supply chains. However, these studies do not consider the impact of government subsidies and adverse weather on sustainable agricultural supply chains.

Fourthly, our work also contributes to the growing stream of research on operations management literature that examines government subsidy. As the government support mechanisms, government subsidies have been studied in different contexts, such as vaccines supply chain [37], renewable energies [38], green technology adoption [39], medicine supply chain [40], and low carbon investment [41]. The most relevant work is to examine the government subsidy in the agricultural supply chain, but only a few papers have studied the agricultural subsidies in the supply chain. Alizamir et al. [2] compare two specific and unique agricultural subsidy mechanisms in the supply chain. Yi and McCarl [16] examine the grain production implications of alternative designs for China’s grain subsidy policy. Unlike these studies, our work focuses on agricultural subsidies under weather-related yield uncertainty.

To the best of our knowledge, this study is the first to model subsidies under weather-related yield uncertainty. Our paper is most closely related to three studies. First, Alizamir et al. [2] examine the impacts of the current price-protection (PLC) subsidy and revenue-protection (ARC) subsidy on different stakeholders, including consumers, the government, and the farming industry in the United States. They develop the models that investigate government subsidy schemes in the U.S. agricultural industry and expand the literature on the intersection of Cournot competition and yield uncertainty. Nevertheless, our work analyzes the impacts of the fixed subsidy and ARC subsidy on different stakeholders in the agricultural industry. Furthermore, we model the interaction between the subsidy programs and environmentally sustainable investment levels under weather-related yield uncertainty. Second, Yi and McCarl [16] investigate the grain production implications of alternative designs for China’s grain subsidy policy. They also examine three subsidy designs including area-based subsidy, quantity-based subsidy, and reduction-cost-based subsidy. However, this study investigates the implications of fixed subsidy and ARC subsidy in the agricultural industry. Meanwhile, we examine the government subsidy by quantitative modeling method. Third, Fu et al. [6] discuss the cooperation mechanism of the agricultural supply chain under weather-related yield uncertainty. But, they do not consider the impact of government subsidy on the different stakeholders in the agricultural industry and characterize the farmer’s environmentally sustainable investment level.

## 3. Problem description and modeling framework

Considering a two-level agricultural supply chain which consists of a farmer and a company. The farmer produces agricultural products which are sold to the company, and in turn the company sells the products to consumers. To ensure the stable supply of healthy agricultural products and the steady increase of farmers’ incomes, government may encourage the farmer to carry out environmentally sustainable agricultural production through economic subsidies.

The environmentally sustainable investment determined by farmers directly affects the yield of agricultural products. We adopt the environmentally sustainable investment level *I* (*I* ∈ [0,1]) to indicate farmers’ effort to ensure the sustainable development of the environment and the production of healthy agricultural products throughout the agricultural production process. Such effort mainly includes several aspects. For example, using chemical fertilizers rationally and pesticides by standards, which is important for reducing soil pollution and ensuring safe production, or refusing to use chemicals such as ripening hormones and refusing to burn straw after harvesting, which is crucial in the safe supply of agricultural products and the reduction of CO_2_ emissions.

In addition, the production process of agricultural products inevitably suffers from uncontrollably adverse weather such as late spring coldness and warm winter, thus the yield of the agricultural products is uncertainty. We consider adverse weather as the main factor affecting the uncertain output of agricultural products and use weather index *w* (such as temperature) to indicate the adverse weather encountered by the farmer in the production process. We assume that the interval $\left(\underline{w},\bar{w}\right)$ represents the suitable weather index for crop growth, where $\bar{w}$ and $\underline{w}$ are the upper bound and the lower bound of the suitable weather index. The interval $\left[\underline{\underline{w}},\bar{\bar{w}}\right]⊃\left(\underline{w},\bar{w}\right)$ is the weather index for having non-disastrous adverse weather, where $\underline{w}$,$\bar{w}$,$\underline{\underline{w}}$, and $\bar{\bar{w}}$ are all uncontrollable exogenous variables. When $w\in \left[\underline{\underline{w}},\underline{w}\right]$, it means that such adverse weather, such as cold spells in late spring and droughts, appears; when $w\in \left[\bar{w},\underline{\underline{w}}\right]$, it means that such adverse weather, such as cold winters and floods, appears. The way to describe adverse weather with a single weather index was widely adopted by privious papers, i.e., Gao et al. [42] and Demirag [43]. Based on the way, this paper, taking the temperature index as an example, studies the impact of weather on the uncertain output of agricultural production.

**Fig 1:**
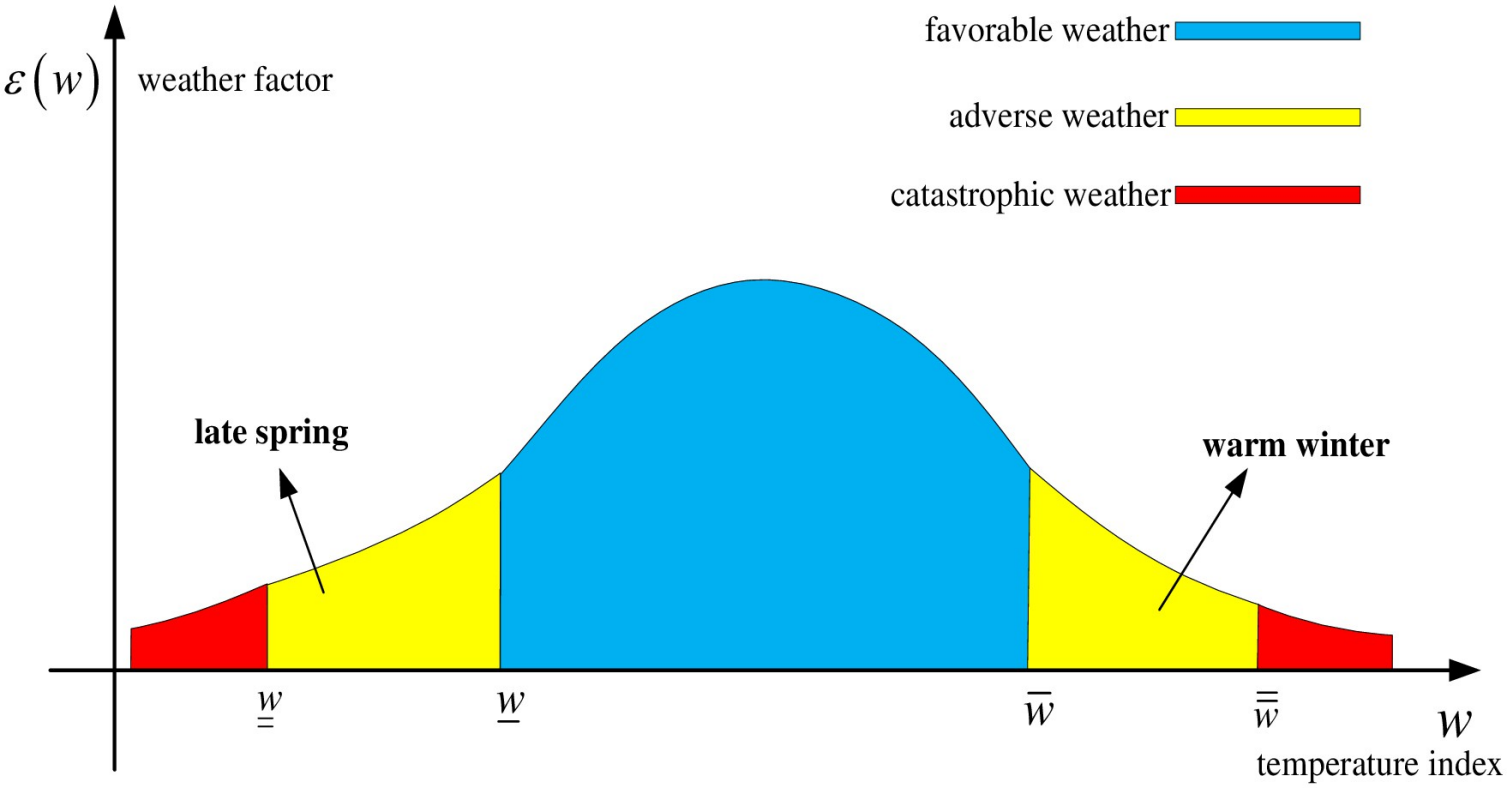
The sketches of the relationship between weather factor and temperature index.

As mentioned early, we assume that the output of agricultural products affected by environmentally sustainable investment (artificially controllable) and temperature indexes (artificially uncontrollable) is *Q*(*I*,*w*) = *q*(*I*)*ε*(*w*), where *q*(*I*) is the output related to the environmentally sustainable investment level *I*, and *ε*(*w*) is the weather factor that influences the output. Note that the environmentally sustainable investment can reduce the use of chemical products, such as pesticides, by some alternative ways which are environmental and thus can improve the outputs. Hence, we assume that *q*(*I*) is a strongly increasing concave function with respect to the environmentally sustainable investment level *I*. Namely, the output of agricultural products increases with the environmentally sustainable investment level and is in marginal decreasing. Moreover, we assume that the probability density function and distribution function of the weather factor *ε*(*w*) in the interval $\left[\underline{\underline{w}},\bar{\bar{w}}\right]$ are *f*(•) and *F*(•) respectively. The multiplicative form for uncertainty yield of agricultural products is widely adopted by previous literature [e.g., 22, 44]. For the same type of agricultural products in a particular region, the expectation and the standard deviation of the weather factor *ε*(*w*) affecting the output are assumed to be *μ* and *σ*, respectively. In addition, the weather conditions in the production process of agricultural products are the common information of companies, farmers, and governments, and all parties can observe specific temperature indexes. Fig 1 depicts the quantitative relationship between weather factors and temperature indexes.

From Fig 1, we can derive some interesting insights. Firstly, when the temperature index satisfies $\underline{w}\leq w\leq \bar{w}$, the weather is suitable for crop growth. Secondly, when the adverse temperature index satisfies $\underline{\underline{w}}\leq w\leq \underline{w}$, the weather factor is positively correlated with the adverse temperature index and the decrease tends to be marginal. That is, the worse the adverse weather, such as late spring coldness, is (i.e., the value of *w* becomes smaller), the lower the yield is. Thirdly, when the adverse temperature index satisfies $\bar{w}\leq w\leq \bar{\bar{w}}$, the weather factor is negatively correlated with the adverse temperature index and the increase tends to be marginal. This means that the worse the adverse weather, such as warm winter, is (the value of *w* becomes larger), the lower the yield is. Finally, when the temperature index satisfies $w<\underline{\underline{w}}$ or $w>\bar{\bar{w}}$, disastrous weather occurs in the process of agricultural production.

We assume that the total cost of the farmer’s environmentally sustainable agricultural production is a second-order differentiable single-increasing convex function of environmentally sustainable investment levels, i.e., *C*(*I*) = *cI*^2^. Such quadratic function is widely adopted by previous literature [15]. In the function, the cost coefficient *c* > 0 indicates that the total cost of farmers’ investment increases with the level of environmentally sustainable investment and is marginally increasing. After the season of agricultural product production, the farmer sells all the agricultural products to the company according to the protection price max {*ω*_*C*_,*ω*}, where *ω*_*C*_ is the guaranteed price determined by the company and *ω* is the actual price in the market, which is uncertain and has the probability density function and probability distribution function *g*(•), *G*(•) in [*L*, *U*]. max {*ω*_*C*_,*ω*} indicates that if the actual price in the market is higher than the guaranteed price agreed, the protection price means that the company will buy the agricultural products from farmers at the actual price. However, if the actual price is lower than the guaranteed price, the company will buy the products at the guaranteed price *ω*_*C*_. *ω*_*F*_ refers to the minimum reserved price determined by farmers. Subsequently, the company processed the agricultural products and sold them at the retail price *p* in the market. At this point, the uncertain market demand faced by the company is *D* which satisfies *D* ∈ [0,+∞), and its probability density function and distribution function are *h*(•) and *H*(•), respectively.

To facilitate the analysis, we assume that both the company and the farmer are rational decision-making bodies, and each seeks to maximize their own interests. Also, we assume that the residual value of the unsold agricultural products and the cost of out-of-stocks are zero. Our paper mainly aims to compare and analyze the impact of different government subsidy formulations on government expenditures, and the performance of companies and farmers. In terms of such target, the optimal decision made by companies and farmers is time-dependent, and their decisions are made based on long-term historical data [2]. Specific quantitative representation of government subsidies is detailed in Section 4.2.1 and Section 4.3.1. For ease of reference, we list the notations this study uses in Table 1.

**Table 1:** List of notations (in order of appearance).

Symbol	Description
*I*	Environmentally sustainable investment level (a decision variable), and I ∈ [0,1]
*w*	Average amount of weather index during the production of agricultural products
$\underline{w}$	Lower bound of the weather suitable for the growth of the crop
$\bar{w}$	Upper bound of the weather suitable for the growth of the crop
* $\bar{\bar{w}}$ *	Upper bound of non-catastrophic adverse weather index
* $\underline{\underline{w}}$ *	Lower bound of non-catastrophic adverse weather index
*Q*(*I*,*w*)	Uncertainty output of agricultural products
*q*(*I*)	Output factor
*ε*(*w*)	Uncertainty weather factor
*f*(•)	PDF function of the weather factor
*F*(•)	CDF function of the weather factor
*μ*	Expectation of the uncertainty weather factor
*σ* ^2^	Variance of the uncertainty weather factor
*C*(*I*)	Farmers’ environmentally sustainable production cost
*c*	Environmentally sustainable production cost coefficient
*ω*	Purchase price in the procurement market (a random variable)
*ω*_*C*_	Guaranteed purchase price decided by the company (a decision variable)
*L*	Lower bound of the purchase price
*U*	Upper bound of the purchase price
*g*(•)	PDF function of the purchase price (random variable *ω*)
*G*(•)	CDF function of the purchase price (random variable *ω*)
*ω*_*F*_	Farmers’ reservation price (an exogenous variable)
*p*	Company’s selling price in the retail market (an exogenous variable)
D	Random market demand (a random variable)
*h*(•)	PDF function of the random market demand *D*
*H*(•)	CDF function of the random market demand *D*
$\pi_{i}^{NS}$	Random profit of *i* (the farmer “F” or the company “C”) under No Subsidy, *i* = *F*,*C*
*k*	Government’s subsidy payment coefficient under Fixed Subsidy
$T_{G}^{FS}$	Government’s total subsidy payment under Fixed Subsidy
$\pi_{i}^{FS}$	Random profit of *i* under Fixed Subsidy, *i* = *F*,*C*
*λ*	Government’s subsidy payment coefficient under ARC Subsidy
$T_{G}^{ARC}$	Government’s total subsidy payment under ARC Subsidy
$\pi_{i}^{ARC}$	Random profit of *i* under ARC Subsidy, *i* = *F*,*C*

## 4 Subsidy policy

In this section, we firstly analyze the optimal sustainable investment decisions of farmers with three models of the non-subsidy formulation (section 4.1), the fixed subsidy formulation (section 4.2), and the Agriculture Risk Coverage (ARC) subsidy formulation (section 4.3), and then investigate the impact of different subsidy formulations and warm winter on costs of governments and profits of the farmer and the company.

### 4.1 No subsidy policy

As a benchmark for comparison with the fixed-subsidy model in section 4.2 and the Agriculture Risk Coverage (ARC) subsidy in section 4.3, we analyze the earnings of farmers and companies under no subsidy formulation in this section firstly. The benchmark for comparative analysis is that when the government does not subsidize agricultural production, both the company and farmers are rational decision-making bodies, and each will make the optimal decision to maximize their interests, with farmers deciding on the optimal environmentally sustainable investment level and companies deciding on the optimal guaranteed price.

#### 4.1.1. Farmer

Without government subsidy, if the output of agricultural products and the purchase price are uncertain, the stochastic profit function of the farmer is as follows.


$$\pi_{F}^{NS}=\text{max}\left\{\omega_{C},\omega \right\}q(I)\varepsilon (w)-cI^{2}$$ (1)

As the expectation of the weather factor is *μ*, and the probability distribution function of the stochastic purchase price of agricultural products in the interval [*L*,*U*] is *G*(·), the farmers’ expected profit function without government subsidy is as follows.


$$E_{w,\omega }\left(\pi_{F}^{NS}\right)=\left(U-\int_{\omega_{c}}^{U}G(\omega )d\omega \right)q(I)\mu -cI^{2}$$ (2)

The second derivative of the Eq (2) above with respect of the environmentally sustainable investment is as follows.


$$\frac{d^{2}E_{w,\omega }\left(\pi_{F}^{NS}\right)}{dI^{2}}=\left(U-\int_{\omega_{C}}^{U}G(\omega )d\omega \right)\mu \frac{d^{2}q(I)}{dI^{2}}-2c$$ (3)

Since the cost coefficient of environmentally sustainable investment is greater than zero, and the output factor is a strongly increasing concave function with respect to the environmentally sustainable investment level, namely, $\frac{d^{2}q(I)}{dI^{2}}\leq 0$, we can obtain $\frac{d^{2}E_{w,\omega }\left(\pi_{F}^{NS}\right)}{dI^{2}}\leq 0$, so that the expected profit of the farmer is a concave function with respect to the environmentally sustainable investment level. Thus, there is an optimal level of environmentally sustainable investment for the farmer to meet the first-order optimal condition, and the optimal level of environmentally sustainable investment determined by the farmer can be obtained by the following function.


$$\left(U-\int_{\omega_{C}}^{U}G(\omega )d\omega \right)\mu \frac{dq(I)}{dI}-2cI=0$$ (4)

#### 4.1.2. Company

Without the government subsidy and in uncertain market demand, the stochastic profit function of the company is as follows.


$$\pi_{C}^{NS}=p\;\text{min}(q(I)\varepsilon (w),D)-\text{max}\left\{\omega_{C},\omega \right\}q(I)\varepsilon (w)$$ (5)

In terms of the analysis of Eq (2), the expected profit function of the company without the government subsidy is as follows.


$$E_{w,\omega ,D}\left(\pi_{C}^{NS}\right)=p\left(q(I)\mu -\int_{0}^{q(I)\mu }H(D)dD\right)-\left(U-\int_{\omega_{C}}^{U}G(\omega )d\omega \right)q(I)\mu$$ (6)

The second derivative of Eq (6) with respect to the guaranteed price is as following function.


$$\frac{d^{2}E_{w,\omega ,D}\left(\pi_{C}^{NS}\right)}{d\omega_{C}^{2}}=-g\left(\omega_{C}\right)q(I)\mu <0$$ (7)

Combined with $\frac{dE_{w,\omega ,D}\left(\pi_{C}^{NS}\right)}{d\omega_{C}}=-G\left(\omega_{C}\right)q(I)\mu <0$, we can obtain $\omega_{C}^{NS^{*}}=\omega_{F}$. A rational company considers its future development when making decisions about purchasing policies, thus it should have positive marginal benefit, that is, the benefit for purchasing unit agricultural products should not be negative, and $\frac{dE_{w,\omega ,D}\left(\pi_{C}^{NS}\right)}{dQ(I,w)}>0$. Based on the analysis of Eq (5), when a company purchases agricultural products, the following constraint condition should be satisfied.


$$p(1-H(q(I)\mu ))>U-\int_{\omega_{C}}^{U}G(\omega )d\omega$$ (8)

When the acquisition constraint equation is satisfied, for the company, the best decision is to take the reserved price of the farmers as the optimal guaranteed purchase price to acquire agricultural products. Thus, combined with the analysis of Eq (4), the optimal level of environmentally sustainable investment determined by farmers should be identified by the following formula.


$$\frac{dq(I)/dI}{I}=\frac{2c}{\left(U-\int_{\omega_{F}}^{U}G(\omega )d\omega \right)\mu }$$ (9)

### 4.2. Fixed subsidy policy

Compared to the no subsidy model in section 4.1, the fixed subsidy formulation refers to a fixed amount paid to the farmer for each kilogram of agricultural product, especially grain production, i.e. production-quantity-based subsidy. To quantify the environmentally sustainable investment with the fixed subsidy formulation, this section will quantitatively describe the fixed subsidy formulation first, and then give the measures of the respective performance of the government, the farmer, and the company under the fixed subsidy policy.

#### 4.2.1. Government

To measure the total government expenditure $T_{G}^{FS}$ under the fixed subsidy policy, we explore the specific characteristics of the fixed subsidy policy first. At present, the fixed subsidy mechanism is mainly applied in developing countries or regions. It provided a direct subsidy for grain producers, including the producers of corn, wheat, and soybeans (farmers, farmers’ professional cooperatives, enterprises, institutions, etc.) [16]. The fixed subsidy provided by governments specifically includes two core elements: first, the total amount of agricultural products produced by farmers, which is measured by the mean value *q*(*I*)*μ* of historical temperature data. Second, the fixed subsidy coefficient of unit agricultural products set by governments, which is recorded as *k*,*k* > 0. Regardless of risk factors such as warm winter during the production of agricultural products, the subsidies paid by the government for the unit of agricultural products remain unchanged. Therefore, with the fixed subsidy formulation, the total government expenditure is as follows.


$$T_{G}^{FS}=kq(I)\mu$$ (10)

#### 4.2.2. Farmer

Similar to the analysis of Eq (1), the stochastic profit function of the farmer under the fixed subsidy policy is as following

$$\pi_{F}^{FS}=\text{max}\left\{\omega_{C},\omega \right\}q(I)\varepsilon (w)-cI^{2}+kq(I)\mu ,$$ (11)

where the first part and the second part are equivalent to the stochastic profit function of farmers without government subsidy in Eq (1), and the third part is the total subsidy paid by the government in Eq (10). Similar to the analysis of Eq (2), the expected profit function of farmers under the fixed subsidy policy is as following.


$$E_{w,\omega }\left(\pi_{F}^{FS}\right)=\left(k+U-\int_{\omega_{C}}^{U}G(\omega )d\omega \right)q(I)\mu -cI^{2}$$ (12)

Similar to the optimal investment level without the government subsidy, the optimal level of environmentally sustainable investment decided by farmers with the fixed subsidy should be determined by the following formula.


$$\frac{dq(I)/dI}{I}=\frac{2c}{\left(k+U-\int_{\omega_{F}}^{U}G(\omega )d\omega \right)\mu }$$ (13)

#### 4.2.3. Company

Since the fixed subsidy is given to the actual producers of crops, the company’s stochastic profit function and the expected profit function are expressed as Eqs (5) and (6) without the government subsidy.

However, in Eqs (5) and (6), we can see that the company’s performance depends not only on the profit function but also on the level of environmentally sustainable investment. In terms of the relationship between the company’s expected profit function and the level of environmentally sustainable investment, combined with the optimal purchase price $\omega_{C}^{FS^{*}}=\omega_{F}$ determined by the company, we can obtain the first derivative of Eq (6) with respect to the environmentally sustainable investment level.


$$\frac{dE_{w,\omega ,D}\left(\pi_{C}^{FS}\right)}{dI}=\left(p(1-H(q(I)\mu ))-\left(U-\int_{\omega_{F}}^{U}G(\omega )d\omega \right)\right)\mu \frac{dq(I)}{dI}$$ (14)

Note from Eq (8) and *d*_*q*_(*I*)/*dI* > 0, we can deduce that $dE_{w,\omega ,D}\left(\pi_{C}^{FS}\right)/dI>0$. That is to say, the company’s expected profit function is positively related to the level of environmentally sustainable investment. The higher the level of environmentally sustainable investment by the farmer, the greater the company’s expected return.

### 4.3. ARC subsidy policy

Unlike the fixed subsidy policy, the Agricultural Risk Coverage (ARC) subsidy policy is one of the main ways the government subsidizes farmers in the Farm Bill signed by US President Obama in 2014 to protect farmers’ incomes from instability. Under ARC subsidy policy, the farmer receives the subsidy when their crop revenue in a given year strop below a reference revenue based on a multiyear moving average of historical crop revenue [2]. In terms of the characterization described by Alizamir et al. [2], we study the optimal level of environmentally sustainable investment under the ARC subsidy policy and the impact of the ARC subsidy policy on farmers and company performance.

#### 4.3.1. Government

To measure the total government expenditure with the ARC subsidy formulation, the typical characteristics of the ARC subsidy policy should be classified. This subsidy policy specifically includes two core elements: first, the income gap, which mainly refers to the difference between the reference revenue and real income *p*_*q*_(*I*)*ε*(*w*) of planting a crop. The reference revenue here refers to the average income over the years, and the expected income *pq*(*I*)*μ* is used to replace the reference revenue. Second, the ARC subsidy factor is set by the government for unit agricultural products, which is recorded as *λ*,*λ* > 0. In the actual production of agricultural products, whether or not adverse weather occurs, the US government sets the subsidy coefficient to a constant value *λ* = 0.85 [2]. To analyze more general situations, we relax the constraint of the fixed subsidy coefficient to be a model parameter, such that analyzing how the ARC subsidy formulation affects the performance of both the company and the farmers. Under the ARC subsidy policy, the total government expenditure is as following.


$$T_{G}^{ARC}=\lambda pq(I)\int_{\underline{\underline{w}}}^{\bar{\bar{\omega }}}\text{max}\left\{0,\mu -\varepsilon (w)\right\}f(\varepsilon (w))d\varepsilon (w)$$ (15)

We analyze the impact of warm winter in the production process of agricultural products, and $w\in \left[\bar{w},\underline{\underline{w}}\right]\subset \left[\underline{\underline{w}},\bar{\bar{w}}\right]$. A similar analysis for late spring coldness (i.e.,$w\in \left[\underline{\underline{w}},\underline{w}\right]\subset \left[\underline{\underline{w}},\bar{\bar{w}}\right]$) in the production process of agricultural products can also be done. When farmers encounter warm winter during the production process, the t subsidy paid by the government is as follows.


$$T_{G}^{ARC}=\lambda pq(I)\int_{\bar{w}}^{\bar{\bar{w}}}\text{max}\left\{0,\mu -\varepsilon (w)\right\}f(\varepsilon (w))d\varepsilon (w)$$ (16)

Based on the characteristic of *ε*(*w*) in Fig 1, we know that, when $\mu >\varepsilon \left(\bar{w}\right)$, the government’s expected expenditure is as follows.


$$E_{w}\left(T_{G}^{ARC}\right)=\lambda pq(I)\left(\mu (F(\varepsilon (\bar{\bar{w}}))-F(\varepsilon (\bar{w})))-(\varepsilon (\bar{\bar{w}})F(\varepsilon (\bar{\bar{w}}))-\varepsilon (\bar{w})F(\varepsilon (\bar{w})))+\int_{\bar{w}}^{\bar{\bar{w}}}F(\varepsilon (w))d\varepsilon (w)\right)$$ (17)

When $u<\varepsilon (\bar{w})$, the government’s expected expenditure is as follows.


$$E_{w}\left(T_{G}^{ARC}\right)=\lambda pq(I)\left(\mu (F(\varepsilon (\bar{\bar{w}}))-F(\mu ))-(\varepsilon (\bar{\bar{w}})F(\varepsilon (\bar{\bar{w}}))-\mu F(\mu ))+\int_{\varepsilon^{-1}(\mu )}^{\bar{\bar{w}}}F(\varepsilon (w))d\varepsilon (w)\right)$$ (18)

#### 4.3.2. Farmer

The stochastic profit function of the farmer under the ARC subsidy policy is as follows

$$\pi_{F}^{ARC}=\text{max}\left\{\omega_{C},\omega \right\}q(I)\varepsilon (w)-cI^{2}+\lambda pq(I)\int_{\bar{w}}^{\bar{\bar{w}}}\text{max}\left\{0,\mu -\varepsilon (w)\right\}f(\varepsilon (w))d\varepsilon (w),$$ (19)

where the first part and the second part are equivalent to the stochastic profit function of farmers in Eq (1) without the government subsidy, and the third part represents the total ARC subsidy from the government. Similar to the analysis of Eqs (2) and (12), the farmer’s expected profit function under the ARC subsidy is as follows.


$$E_{w,\omega }\left(\pi_{F}^{ARC}\right)=\left(U-\int_{\omega_{C}}^{U}G(\omega )d\omega \right)q(I)\mu -cI^{2}+\lambda pq(I)\left(\mu (F(\varepsilon (\bar{\bar{w}}))-F(x))-(\varepsilon (\bar{\bar{w}})F(\varepsilon (\bar{\bar{w}}))-xF(x))+\int_{\varepsilon^{-1}(x)}^{\bar{\bar{w}}}F(\varepsilon (w))d\varepsilon (w)\right)$$ (20)

When $\mu >\varepsilon \left(\bar{w}\right)$, $x=\varepsilon \left(\bar{w}\right)$; when $\mu <\varepsilon \left(\bar{w}\right)$, *x* = *μ*. Similar to the solution of the optimal level under the fixed subsidy policy, the optimal level of environmentally sustainable investment decided by farmers should be determined by the following formula.


$$\frac{dq(I)/dI}{I}=\frac{2c}{\lambda p\left(\mu (F(\varepsilon (\bar{\bar{w}}))-F(x))-(\varepsilon (\bar{\bar{w}})F(\varepsilon (\bar{\bar{w}}))-xF(x))+\int_{\varepsilon^{-1}(x)}^{\bar{\bar{w}}}F(\varepsilon (w))d\varepsilon (w)\right)+\left(U-\int_{D_{F}}^{U}G(\omega )d\omega \right)\mu }$$ (21)

#### 4.3.3. Company

Similar to the analysis in Section 4.2.2, the stochastic profit function $\pi_{C}^{ARC}$ and the expected profit function $E_{w,\omega ,D}\left(\pi_{C}^{ARC}\right)$ of the company under the ARC subsidy policy is expressed as the functions without the government subsidy in Eqs (5) and (6). That is, the government’s provision of the ARC subsidy will not change the company’s profit function expression. However, as the ARC subsidy affects the level of environmentally sustainable investment, combined with the analysis of the Eq (14) above, the ARC subsidy will improve the company’s performance by boosting the level of sustainable investment.

## 5. Subsidy policy comparison and implications

Section 4 presents an equilibrium analysis of the level of environmentally sustainable investment under three models (no subsidy, fixed subsidy, and ARC subsidy). To better compare and analyze the implementation effects and implementation conditions of the different subsidy models, we conduct a two-by-two analysis of the three subsidies in this section to explore the differences in the impact of varying government subsidy schemes on the level of environmentally sustainable investment and the performance of other stakeholders (government, farmers, and companies). Specifically, the differences between the fixed and no subsidy models are compared in section 5.1, the ARC subsidy model is compared with the no subsidy model in section 5.2, and the fixed and ARC subsidy models are compared in section 5.3.

### 5.1. Comparison of fixed subsidy and no subsidy

Compared with the benchmark, it is worthy of an in-depth study of whether the fixed subsidy formulation provided by the government can encourage farmers to improve the level of environmentally sustainable investment. Proposition 1 below depicts a comparative analysis of the environmentally sustainable investment level with the two formulations.

**Proposition 1.** *For given* $w\in \left[\bar{w},\bar{\bar{w}}\right]$, $I_{F}^{FS^{*}}$ *and* $I_{F}^{NS^{*}}$ *decrease with aggravation of the adverse weather of warm winter, and satisfy* $I_{F}^{FS^{*}}>I_{F}^{NS^{*}}$.

**Proof of Proposition 1.** Based on the analysis methods of Brânzei et al. [45] and Fu et al. [6], the mixed partial derivative of $\pi_{F}^{NS}$ concerning (*I*,*w*) is solved, and we obtain $\partial^{2}\pi_{F}^{NS}/\partial I\partial w<0$. It means that $\pi_{F}^{NS}$ satisfies the submodular to (*I*,*w*), so that $I_{F}^{NS^{*}}$ is the decreasing function of *w*. Similarly, $I_{F}^{FS^{*}}$ can be demonstrated as the decreasing function of *w*.

Eqs (9) and (13) have given the optimal sustainable investment level with the two subsidy formulations. To demonstrate $I_{F}^{FS^{*}}>I_{F}^{NS^{*}}$, the monotonicity of $\theta (I)=\frac{dq(I)/dI}{I}$ concerning the level of environmentally sustainable investment needs to be identified. Thus, the first derivative of *θ*(*I*) concerning the environmentally sustainable investment level is as follows.


$$\frac{d\theta (I)}{dI}=\frac{1}{I^{2}}\left(I\frac{d^{2}q(I)}{dI^{2}}-\frac{dq(I)}{dI}\right)$$ (22)

Since *d*^2^*q*(*I*)/*dI*^2^ < 0 and *dq*(*I*)/*dI* > 0, we obtain *dθ*(*I*)/*dI*. It means that *θ*(*I*) is the monotone decreasing function of *I*. Combined with *kμ* > 0, Eqs (9) and (13), $I_{F}^{FS^{*}}>I_{F}^{NS^{*}}$ is demonstrated. Q.E.D

Proposition 1 shows that regardless of the provision of government subsidies, the occurrence of adverse weather of warm winter will reduce the optimal level of environmentally sustainable investment. On the other hand, Proposition 1 also shows that the government’s approach to providing the fixed subsidy to the farmer can indeed motivate it to increase sustainable investment. Although agricultural production is inevitably affected by adverse weather such as warm winter, the fixed subsidy provided by the government can weaken the adverse effects to a certain extent.

The government’s provision of subsidies is not only to ensure the sustainable development of the agricultural environment but also to increase the income of farmers. Therefore, it is necessary to analyze the impact of the fixed subsidy policy on the income of farmers and the company. Combined with the Eqs (2) and (12), in the same weather and sustainable investment level, since *kq*(*I*)*μ* > 0, the fixed subsidy policy will certainly increase the farmer’s income, and $\pi_{F}^{FS}>\pi_{F}^{NS}$. As for the impact of the fixed subsidy policy on the company’s income, the analysis in Section 4.2.3 shows that the company’s expected profit function is positively correlated with the level of environmentally sustainable investment. Proposition 1 shows $I_{F}^{FS^{*}}>I_{F}^{NS^{*}}$, so that $\pi_{C}^{FS}>\pi_{C}^{NS}$. Thus, the following proposition 2 can be deduced.

**Proposition 2.** *For given* $w\in \left[\bar{w},\bar{\bar{w}}\right]$, *the profits of the farmer and the company under the fixed subsidy policy satisfy* $\pi_{F}^{FS}>\pi_{F}^{NS}$, $\pi_{C}^{FS}>\pi_{C}^{NS}$.

**Fig 2:**
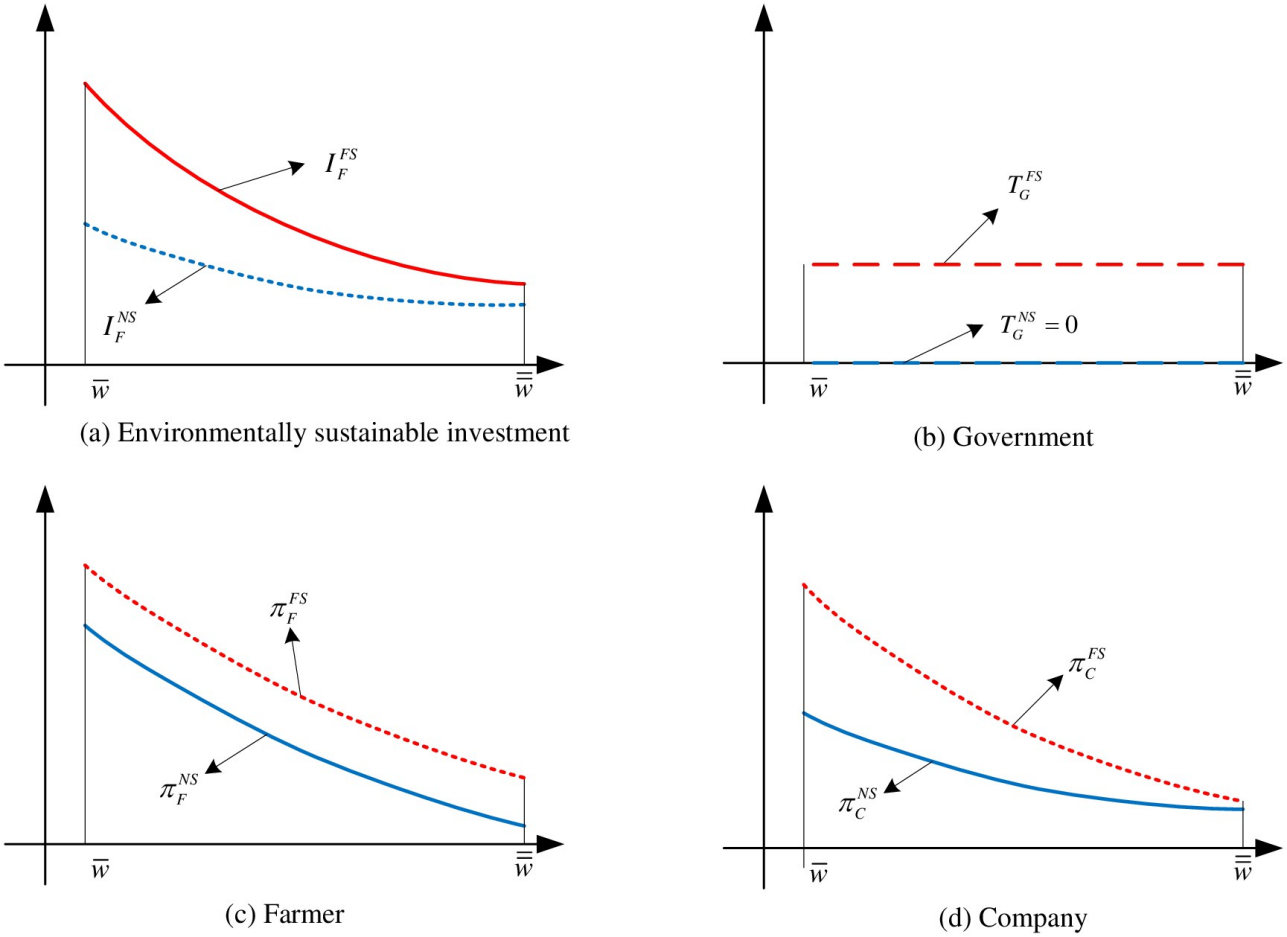
The sketches of comparison of fixed subsidy and no subsidy. (a) Environmentally sustainable investment, (b) Government, (c) Farmer, (d) Company.

Combined with Proposition 1, although the farmer’s environmentally sustainable investment decreases with adverse warm weather increasing, the fixed subsidy policy can effectively increase their level of environmentally sustainable inputs as numerically shown in Fig 2(a). The governments only need to pay fixed subsidy based on the expected output of agricultural products in Fig 2(b), and there is no additional cost. According to Proposition 2, the fixed subsidy policy can effectively improve the earnings of farmers as numerically shown in Fig 2(c). Meanwhile, the fixed subsidy policy is also effective in improving the earnings of both companies as numerically shown in Fig 2(d), it also indicates that the government’s fixed subsidy mechanism can achieve a win-win result for both the farmer and the company. But compared to farmers, the company’s profit improved even less as the warm winter aggravating.

Fixed subsidy policy which is widely used in developing countries (such as China’s fixed subsidy for grain) have played a positive role in ensuring farmers’ incomes and increasing environmentally sustainable investment. However, some problems should also be solved in the operation of this policy. For example, the fixed subsidy policy is based on the production and do not consider the risk factors in the agricultural production chain, such as the uncertainty of farmers’ income caused by adverse weather. Thus, we continue to explore the role that ARC subsidy policy which is widely used in developed countries.

### 5.2. Comparison of ARC subsidy and no subsidy

In contrast to the benchmark, this section studied whether the government-provided ARC subsidy policy can increase the level of environmentally sustainable investments. Referring to the analysis of Proposition 1, $I_{F}^{ARC^{*}}$ can be proved as a decreasing function with respect to warm winter. Combined with the description of the level of environmentally sustainable investments in Eqs (9) and (21) and $\lambda pq(I)\int_{\bar{w}}^{\bar{\bar{w}}}\text{max}\left\{0,\mu -\varepsilon (w)\right\}dF(\varepsilon (w))>0$, we can obtain $I_{F}^{ARC^{*}}>I_{F}^{NS^{*}}$. Thus, referring to the proof of Proposition 1, Proposition 3 can be deduced directly.

**Proposition 3.** *For given* $w\in \left[\bar{w},\bar{\bar{w}}\right]$, $I_{F}^{ARC^{*}}$ and $I_{F}^{NS^{*}}$ *decrease with the aggravation of warm winter, and satisfy* $I_{F}^{ARC^{*}}>I_{F}^{NS^{*}}$.

From Proposition 3, we find a core conclusion which is similar to Proposition 1: The ARC subsidy policy can weaken the adverse effects of warm winter to a certain extent, and effectively increase the environmentally sustainable investment level. We should further answer the following question: what is the impact of the ARC subsidy formulation on the profit of both the farmer and the company? Based on the concept of the ARC subsidy design, for a given warm winter condition, we can obtain $\lambda pq(I)\int_{\bar{w}}^{w}\text{max}\left\{0,\mu -\varepsilon (w)\right\}dF(\varepsilon (w))>0$. Thus, the ARC subsidy policy can increase the income of the farmer comparing with that under benchmark, i.e., $\pi_{F}^{ARC}>\pi_{F}^{NS}$. For company, as the company’s revenue is positively correlated with the environmentally sustainable investment level, referring to the analysis of Proposition 3, we deduce $\pi_{C}^{ARC}>\pi_{C}^{NS}$. Thus, Proposition 4 can be obtained.

**Proposition 4.** *For given* $w\in \left[\bar{w},\bar{\bar{w}}\right]$, *the income of the farmer and the company under the ARC subsidy policy satisfy* $\pi_{F}^{ARC}>\pi_{F}^{NS}$ and $\pi_{C}^{ARC}>\pi_{C}^{NS}$.

**Fig 3:**
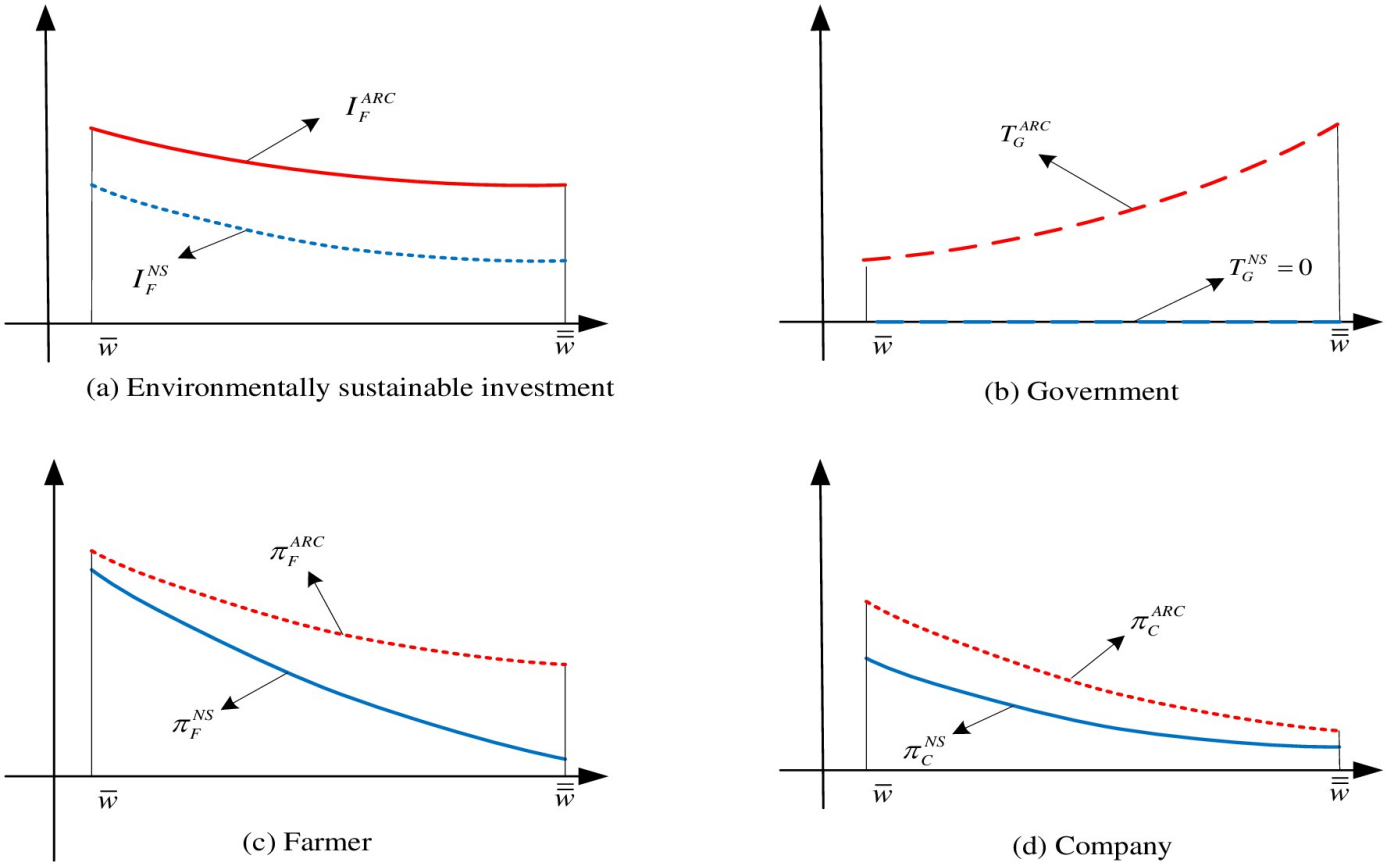
The sketches of comparison of ARC subsidy and no subsidy. (a) Environmentally sustainable investment, (b) Government, (c) Farmer, (d) Company.

Combined with Proposition 3 and Proposition 4, a conclusion similar to the fixed subsidy analysis in Section 5.1 is found, that is, the government-provided ARC subsidy policy can effectively improve the level of environmentally sustainable investment which is illustrated in Fig 3(a). However, the amount of subsidy provided by the government is determined according to the risks faced by the farmer, and the subsidy increases with the aggravation of adverse weather, which is illustrated in Fig 3(b). From this point, the ARC subsidy formulation is more advantageous. However, it should not be overlooked that the ARC subsidy policy increases the government’s additional management costs, such as the measurement and audit of farmers’ income under the influence of adverse weather of warm winter. Proposition 3 also shows that the ARC subsidy mechanism can effectively improve the earnings of farmers (see Fig 3(c)), and with the aggravation of warm winter, the farmer’s income improves more. In addition, the ARC subsidy policy also can effectively increase the company’s profit, which means that the government’s ARC subsidy mechanism can achieve a win-win result for both the farmer and the company. But the company’s earnings will increase even less as severe weather aggravating (see Fig 3(d)).

Generally, both the fixed subsidy policy which is widely used in developing countries and the ARC subsidy policy which is widely applied in developed countries can increase the level of environmentally sustainable investment and achieve a win-win result for both the farmer and the company. However, specifically, which subsidy policy is better for the farmer? What are the boundary conditions for farmers to choose the subsidy formulation? To answer these questions, a detailed comparative analysis of the two subsidy formulations is needed.

### 5.3. Comparison of fixed subsidy and ARC subsidy

Based on both Eqs (13) and (21), we find that the optimal level of environmentally sustainable investments under the fixed subsidy policy and the ARC subsidy policy depends on the distribution of agricultural product purchase prices and the distribution of adverse weather of warm winter. The comparison of the income or expenditure of various stakeholders (farmers, companies, and governments) under the two subsidy policies is strictly dependent on the optimal level of environmentally sustainable investment. Therefore, to further analyze and obtain effective results, this section will analyze the impact of fixed subsidy policy and ARC subsidy policy on stakeholders and the impact of model parameters on these subsidy policies.

Referring to the quantitative description of functional forms from Fu et al. [6], we assumed that the output factor of farmers producing agricultural products is *q*_0_*I*^1/2^; the weather factors faced by farmers in the production process of agricultural products follow a uniform distribution in the warm winter index range $\left[\bar{w},\bar{\bar{w}}\right]$, namely, $\varepsilon (w)\sim U(\varepsilon (\bar{w}),\varepsilon (\bar{\bar{w}}))$; the volatile market purchase price faced by farmers is subject to a uniform distribution, namely, *w* ~ *U*(0,4); the stochastic market demand distribution faced by companies satisfies *D* ~ *U*(0,*D*_0_); other relevant model parameters are set to *ω*_*F*_ = 2 and *p* = 5. Similar to the analysis of the optimal level of the environmentally sustainable investment in Proposition 1 and Proposition 3, we can get Proposition 5.

**Proposition 5.** *For given* $w\in \left[\bar{w},\bar{\bar{w}}\right]$, *when* $0\leq F(\varepsilon (w))<\frac{2k}{5\lambda }$, $I_{F}^{FS^{*}}>I_{F}^{ARC^{*}}$; *when* $\frac{2k}{5\lambda }\leq F(\varepsilon (w))\leq 1$, $I_{F}^{FS^{*}}\leq I_{F}^{ARC^{*}}$.

**Proof of Proposition 5.** Based on the model parameters of the above propositions, similar to the analysis of Proposition 1, the optimal level of sustainable investment with the government’s fixed subsidy formulation is determined by the following formula.


$$\frac{q_{0}}{2}I^{-\frac{3}{2}}=\frac{2c}{\left(k+\frac{5}{2}\right)\mu }$$ (23)

The optimal level of sustainable investment under the ARC subsidy can be determined by the following formula.


$$\frac{q_{0}}{2}I^{-\frac{3}{2}}=\frac{2c}{\frac{5}{2}\mu +5\lambda F(\varepsilon (w))\left(u-\frac{1}{2}(\varepsilon (\bar{w})+\varepsilon (\bar{\bar{w}}))\right)}$$ (24)

Without loss of generality, we assumed $u=(\varepsilon (\bar{w})+\varepsilon (\bar{\bar{w}}))$, so that the denominator of the Eq (24) is $\frac{5}{2}(\mu +\lambda uF(\varepsilon (w)))$. Combined with the analysis of Eq (23), when $0\leq F(\varepsilon (w))<\frac{2k}{5\lambda }$, $\left(k+\frac{5}{2}\right)\mu >\frac{5}{2}(\mu +\lambda uF(\varepsilon (w)))$. Since $I^{-\frac{3}{2}}$ is the monotone decreasing function of *I*, $I_{F}^{FS^{*}}>I_{F}^{ARC^{*}}$ is obtained. Similarly, when $\frac{2k}{5\lambda }\leq F(\varepsilon (w))\leq 1$, $I_{F}^{FS^{*}}\leq I_{F}^{ARC^{*}}$ is obtained. Q.E.D

**Fig 4:**
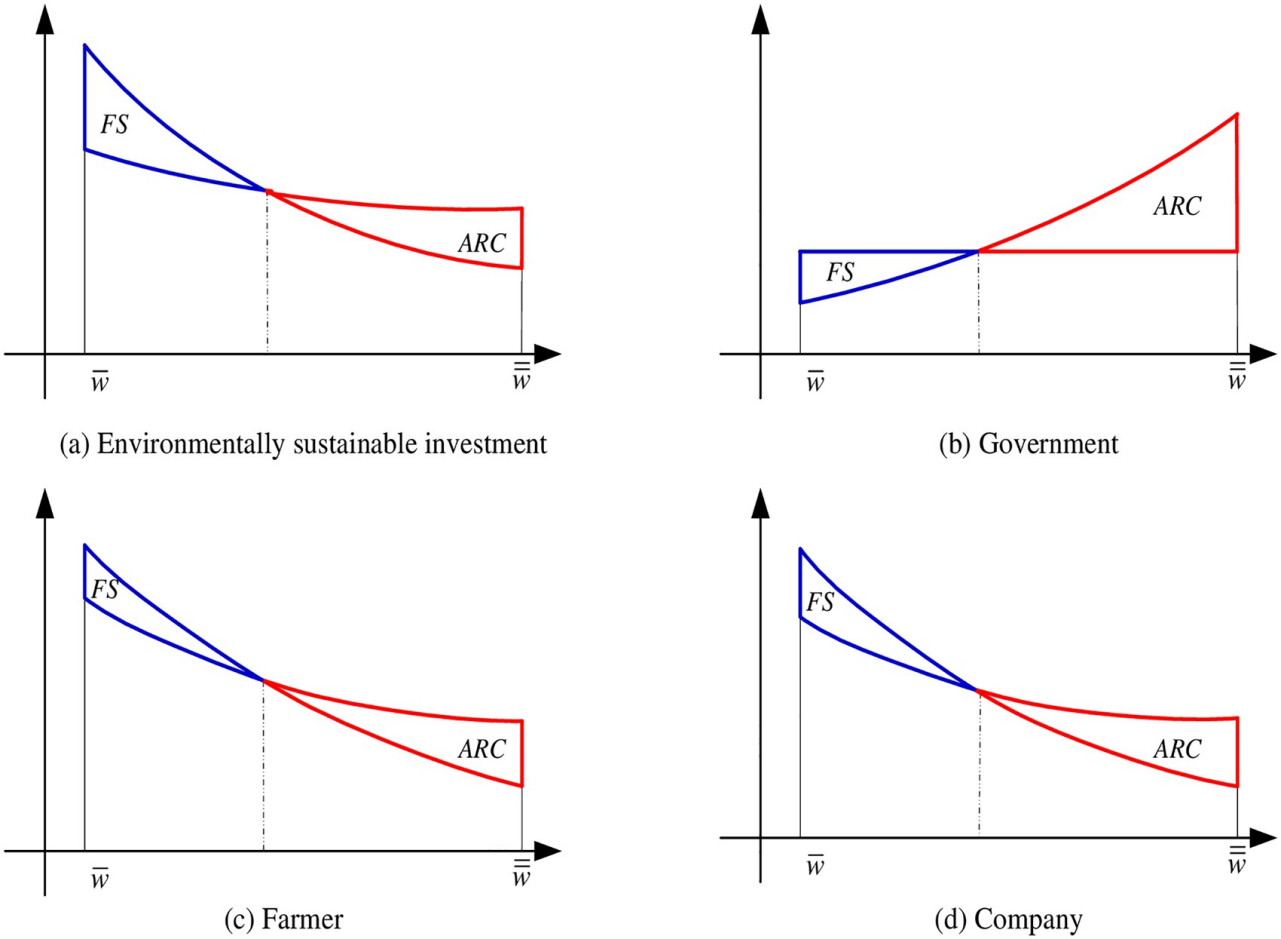
The sketches of subsidy regions. (a) Environmentally sustainable investment, (b) Government, (c) Farmer, (d) Company.

Proposition 5 reveals the impact of different subsidy policies on the level of environmentally sustainable investments in adverse weather of warm winter. Proposition 5 shows that the advantage of the fixed subsidy policy gradually disappears with the aggravation of adverse weather of warm winter. When the adverse weather of warm winter exceeds a certain level (the weather index satisfies *F*(*ε*(*w*) = 2*k*/5*λ*), the ARC subsidy policy is better than the fixed subsidy policy in encouraging the farmer to increase the level of environmentally sustainable investment. with the aggravation of adverse weather of warm winter, the optimal level of environmentally sustainable investment under the two subsidy policies will continue to decline. However, compared with the fixed subsidy policy, the ARC subsidy policy has a significant advantage in encouraging the farmer to increase the level of environmentally sustainable investment (see Fig 4(a)).

**Preposition 6.** *For given $w\in \left[\bar{w},\bar{\bar{w}}\right]$, the benefit or payment of each stakeholder (farmers, companies, governments) under the fixed subsidy policy and the ARC subsidy policy satisfies*

- *当$0\leq F(\varepsilon (w))<\frac{2k}{5\lambda }$*, $\pi_{F}^{FS}\geq \pi_{F}^{ARC}$, $\pi_{C}^{FS}\geq \pi_{C}^{ARC}$, $T_{G}^{FS}\geq T_{G}^{ARC}$;
- *当$\frac{2k}{5\lambda }\leq F(\varepsilon (w))\leq 1$*, $\pi_{F}^{FS}<\pi_{F}^{ARC}$, $\pi_{C}^{FS}<\pi_{C}^{ARC}$, $T_{G}^{FS}<T_{G}^{ARC}$。

**Proof of Preposition 6.** (i) When $0\leq F(\varepsilon (w))<\frac{2k}{5\lambda }$, from Proposition 5, we know $I_{F}^{FS^{*}}>I_{F}^{ARC^{*}}$. Similar to the analysis of Proposition 2 and Proposition 4, $\pi_{F}^{FS}\geq \pi_{F}^{ARC}$ and $\pi_{C}^{FS}\geq \pi_{C}^{ARC}$ are obtained. Based on a given model parameter and Eq (10), the government’s expenditure with the fixed subsidy is $T_{G}^{FS}=kuq_{0}I^{\frac{1}{2}}$. Similarly, based on Eq (17), the government’s expenditure with the ARC subsidy is $T_{G}^{ARC}=\frac{5}{2}\lambda uq_{0}I^{\frac{1}{2}}F(\varepsilon (w))$. When $0\leq F(\varepsilon (w))<\frac{2k}{5\lambda }$, $T_{G}^{FS}<T_{G}^{ARC}$ is obtained.

(ii) is similar to (i), and when $\frac{2k}{5\lambda }\leq F(\varepsilon (w))\leq 1$, $\pi_{F}^{FS}<\pi_{F}^{ARC}$, $\pi_{C}^{FS}<\pi_{C}^{ARC}$ and $T_{G}^{FS}<T_{G}^{ARC}$ are obtained. Q.E.D

Proposition 6 reveals the impact of the fixed subsidy policy and the ARC subsidy policy on stakeholders when facing warm winter adverse weather. We found that when the adverse impact from a warm winter is weaker, the government provides more subsidies under the fixed subsidy policy compared to the ARC subsidy policy which is illustrated in Fig 4(b). In this case, both the company and the farmer will gain higher profits as shown in Fig 4(c) and 4(d). The reason is in line with the fact that the risk of loss to the farmer from adverse warm weather is lower at this time. Therefore, the subsidy provided by the government under the ARC subsidy policy is lower than that under fixed subsidy policy. In worse adverse weather, when the adverse weather exceeds a certain level (the weather index satisfies *F*(*ε*(*w*) = 2*k*/5*λ*), the farmer’s earnings will be lower (see Fig 4(c)), also the company’s profit (see Fig 4(d)). However, the advantages of ARC subsidy policy will gradually emerge, and the more severe the adverse warm weather, compared to the fixed subsidy policy, the more obvious the advantages of the ARC subsidy scheme will be, and the farmers and companies will gain higher profits as a result.

## 6. Conclusions

In contrast with Alizamir et al [2] who studied the impact of current price protection (PLC) subsidy policy and income protection (ARC) subsidy policy on different stakeholder, we investigate the impact of fixed and ARC subsidy policies on different stakeholders in the agricultural industry. In addition, we analyze the interaction between different government subsidy policy and the level of environmentally sustainable investment under weather-related yield uncertainty. Next, different from Yi and McCarl [16], who study the impact of alternative designs of grain subsidy policies on grain production in China. This study explores the impact of fixed subsidies and ARC subsidies on sustainable agricultural supply chains. Also, we analyze three different government subsidy models through a quantitative modelling approach. Finally, differently from the study by Fu et al [6] who discuss the mechanisms of agricultural supply chain cooperation under weather-related yield uncertainty, we consider the impact of government subsidies on different stakeholders in the agricultural industry and characterize farmer’s level of environmentally sustainable investment.

This paper studies the environmentally sustainable investment of a two-level agricultural supply chain consisting of an individual farmer and a company with government subsidy. Taking the adverse weather of warm winter as an example, we give the optimal level of environmentally sustainable investment under the three models of no subsidy, the fixed subsidy and the ARC subsidy, with the condition of the uncertain output of weather-related agricultural products. This study also compares and analyzes the income of the farmer and the company and the cost of governments under different subsidy policy.

In this study, we find several interesting observations and useful policy implications for the environmentally sustainable operation of the agricultural supply chain with government subsidy. First, in the adverse weather of warm winter, compared with no subsidy policy, the fixed subsidy policy and the ARC subsidy policy can encourage the farmer to increase their environmentally sustainable investments. If the adverse weather of warm winter is not relatively serious, the fixed subsidy policy has a significant advantage in encouraging the farmer’s environmentally sustainable investments. Otherwise, if adverse weather of warm winter aggravates to a certain degree, the ARC subsidy policy is better than the fixed subsidy policy in encouraging the farmer’s environmentally sustainable investments. Second, under the influence of adverse weather of warm winter, both the fixed subsidy and the ARC subsidy can increase the incomes of the farmer and the company. The aggravation of adverse weather inevitably increases government expenditures, and which policy has better performance depends on the relationship of the warm winter index and the subsidy coefficient. Third, for the farmer, the fixed subsidy policy is preferred when the adverse weather is weak, otherwise, the ARC subsidy policy is preferred when the adverse weather is strong. Finally, from the perspective of the agricultural subsidy designer, although the fixed subsidy policy is not targeted to weaken the risk loss faced by the farmer, it is of low executable cost, in no need of monitoring the moral hazard of farmers. Thus, developing countries with relatively weak regulatory capabilities often adopt fixed subsidy policy, while developed countries with strong regulatory capabilities often choose ARC subsidy policy. From the perspective of beneficiaries of agricultural subsidy, the ARC subsidy policy is more beneficial for the farmer if agricultural production is expected to suffer from adverse weather. In the operation of environmentally sustainable agriculture, farmers are in a vulnerable position. As long as the government provides subsidies, farmers will increase the environmentally sustainable investment level. If the government offers farmers opportunities to choose, they will choose the subsidy policy based on their own risk preferences, subsidy factors of various subsidy policy, and uncontrollable adverse weather.

This study still has some limitations. To better analyze the impact of government subsidy on the sustainable operation of the agricultural environment, we simplify the model and only consider the environmentally sustainable investment of a individual farmer. Therefore, it would be meaningful for future studies to explore environmentally sustainable investments under competition from multiple homogeneous or heterogeneous farmers under government subsidy. In addition, the risk preferences of farmers (risk-averse, risk-neutral, and risk-loving) influence their optimal environmentally sustainable input decisions, hence it would be a more interesting research direction to study the issue of environmentally sustainable inputs under government subsidy taking into account the risk preferences of farmers.
